# Amelioration of Chronic Ethanol Administration‐Induced Learning and Memory Impairments by High‐Intensity Interval Training (HIIT) and Ritalin

**DOI:** 10.1002/brb3.70539

**Published:** 2025-05-08

**Authors:** Sara Shirazpour, Farahnaz Taheri, Gholamreza Sepehri, Mahla Zangiabadizadeh, Mostafa Zangiabadi, Najmeh Sadat Hosseini, Sara Sheikhi, Azadeh Shahrokhi Raeini, Sara Sheibani Tezerji

**Affiliations:** ^1^ Institute of Neuropharmacology Neuroscience Research Center Kerman University of Medical Sciences Kerman Iran; ^2^ Neurology Research Center, Institute of Neuropharmacology Kerman University of Medical Sciences Kerman Iran; ^3^ Veterinary Medicine Student, School of Veterinary Medicine Shahid Bahonar University of Kerman Kerman Iran; ^4^ Department of Behavioral and Molecular Neurobiology Regensburg Center for Neuroscience University of Regensburg Regensburg Germany; ^5^ Physiology Research Center Institute of Neuropharmacology, Kerman University of Medical Sciences, Kerman Iran; ^6^ Bone Vascular and Microcirculation Laboratory Department of Kinesiology, University of Texas at Arlington, Arlington, TX, 76019 USA

**Keywords:** ethanol, high‐intensity interval training (HIIT), learning and memory, oxidative stress, Ritalin

## Abstract

**Objectives:**

The current study aimed to investigate the impacts of 8‐week high‐intensity interval training (HIIT) and Ritalin (RIT), alone and in combination, on cognitive functions and hippocampal oxidative parameters following chronic ethanol consumption in male rats.

**Methods:**

A total of 56 adult male rats were divided into 8 groups and received one of the following treatments: ethanol 20% (ET) (3 mL/kg/day, orally, 5 consecutive days/week in weeks 1–4, and 3 consecutive days/week in weeks 4–8), RIT (10 mg/kg, intraperitoneally, three consecutive times/week for 8 weeks), HIIT + SAL (five consecutive times/week for 8 weeks + saline injection), or saline (1 mL/day, intraperitoneally, three consecutive times/week for 8 weeks). Cognitive performance was assessed using the Morris water maze (MWM) and passive avoidance tasks. Oxidative stress markers, including malondialdehyde (MDA), glutathione peroxidase (GPx), and total antioxidant capacity (TAC), were measured in the hippocampus using thiobarbituric acid reactive substances (TBARS) and ferric reduction antioxidant power (FRAP). Nitric oxide (NO) level in the hippocampus was determined using an NO Assay Kit (Natrix, Arman Biotech, Iran).

**Results:**

Chronic ethanol administration impaired cognitive functions. However, RIT, HIIT, and their combination significantly improved these impairments. Furthermore, RIT increased ethanol‐induced oxidative stress, whereas HIIT reduced it, even in the combination group.

**Conclusion:**

Chronic ethanol consumption caused learning and memory deficits and disrupted oxidant/antioxidant balance in the hippocampus of rats. HIIT potentially improved memory impairments by restoring this balance, whereas RIT ameliorated cognitive dysfunction through a mechanism that requires further investigation.

## Graphical abstract

The current study investigated the impacts of 8‐week high‐intensity interval training (HIIT) and Ritalin, alone and in combination, on cognitive functions and hippocampal oxidative parameters following chronic ethanol consumption in male rats. Adult male rats were divided into eight groups and received one of the following treatments: ethanol 20% (ET) (3 mL/kg/day, orally, 5 consecutive days/week in weeks 1–4, and 3 consecutive days/week in weeks 4–8), Ritalin (RIT) (10 mg/kg, intraperitoneally, three consecutive times/week for 8 weeks), HIIT + SAL (five consecutive times/week for 8 weeks + saline injection), or saline (1 mL/day, intraperitoneally, three consecutive times/week for 8 weeks). Cognitive performance was assessed using the Morris water maze (MWM) and passive avoidance tasks. Oxidative stress markers were measured in the hippocampus. Chronic ethanol consumption caused learning and memory deficits and disrupted oxidant/antioxidant balance in the hippocampus of rats. HIIT potentially improved memory impairments by restoring this balance, whereas Ritalin ameliorated cognitive dysfunction through a mechanism that requires further investigation.

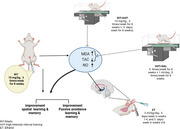

## 1 Introduction

Ethanol consumption is considered a major concern by the World Health Organization (WHO), as it causes nearly 3.3 million deaths annually, accounting for 5.9% of all deaths worldwide (World Health Organization 2019). Ethanol is one of the most prevalent factors leading to poisoning in the central nervous system (CNS) and has irreversible effects (Patil et al. 2015).

Cognitive function plays a crucial role in the quality and length of life, with learning and memory being vital components of cognition (Dyrbye et al. 2017). Clinical studies have revealed that alcohol consumption may affect cognitive functions such as attention, memory, decision‐making, planning, and learning ability (Crews et al. 2016). Long‐term and excessive alcohol consumption can lead to reduced cognitive performance (Zahr and Pfefferbaum 2017) and is recognized as a significant risk factor for dementia (Langballe et al. 2015). Additionally, alcohol causes structural damage to the brain.

The hippocampus plays a key role in learning and memory formation (Miguez et al. 2015). Notably, individuals consuming over 240 g/week of alcohol have a higher risk of structural damage, particularly in the hippocampus (Topiwala et al. 2017). Moreover, even moderate alcohol consumption is associated with adverse brain outcomes, including hippocampal atrophy (Topiwala et al. 2017). Moderate alcohol intake in older individuals has been linked to reduced total brain volume (Paul et al. 2008), increased ventricle size (Ding et al. 2004), gray matter atrophy (Mukamal et al. 2001), and reduced density of frontal and parietal gray matter (Sachdev et al. 2008).

Animal studies have revealed neuronal cell death and inflammation in the brains of rats following ethanol administration (Mohseni et al. 2021). Alcohol exposure during the first 10 postnatal days in rats, which mimics ethanol consumption during the last gestational trimester in humans, increased acetylcholinesterase (Ach) activity and resulted in memory impairments (Bariselli et al. 2023). Moreover, Jiao et al. demonstrated memory impairments caused by long‐term ethanol intake in rats, which may be related to the regulation of hippocampal dysfunction (Jiao et al. 2023).

Studies have shown that 4 days of ethanol drinking reduced neurogenesis and the expression of brain‐derived neurotrophic factor (BDNF) in the hippocampal region of rats (Feizolahi et al. 2019). Recent research has indicated that long‐term ethanol exposure impairs, learning and memory function by altering the density and morphology of dendritic spines in the brain (Pascual et al. 2021) and exacerbating oxidative stress (Akbari et al. 2023). Furthermore, acute ethanol administration decreased antioxidant levels in rats (Airaodion et al. 2019).

Oxidative stress occurs due to an imbalance between oxidants and antioxidants, resulting in the accumulation of reactive oxygen species (ROS) (Kregel and Zhang 2007). Previous studies have demonstrated deficits in learning and memory caused by increased oxidative stress induced by aging or Alzheimer's disease (Ionescu‐Tucker and Cotman 2021; Hao et al. 2014). Many studies have reported elevated brain oxidative stress in response to ethanol consumption (Gil‐Mohapel et al. 2019; Imran et al. 2020). Therefore, protective effects against oxidative stress are crucial to delaying cerebral aging and improving memory performance. Moreover, various drugs, such as RIT, have shown positive effects on cognitive function.

RIT is a drug that enhances cognitive performance, particularly attention and cognitive control, and is often used to improve memory (Colzato and Arntz 2017). Interestingly, several studies have reported improved memory in healthy adults following RIT consumption (Peres et al. 2023). The previous research has shown that RIT ameliorates the spatial memory impairments caused by an increase in Ach in the prefrontal cortex of rats (Scherer et al. 2010). Furthermore, chronic RIT consumption facilitates the regulation of dopamine‐inducing genes by increasing the expression of genes involved in modulating the serotonergic system (Daniali et al. 2013).

Physical activities, particularly exercise, have been shown to neuroprotective effects. These include reducing oxidative damage (Radak et al. 2013), modulating neurotransmitter levels (Subramanian et al. 2014; Maddock et al. 2016), decreasing brain inflammation (Lin et al. 2020), promoting neuroplasticity (Zhao et al. 2020), and improving behavioral performance (Rodrigues et al. 2020). Exercise also enhances memory by stimulating neurogenesis (Epp et al. 2021), angiogenesis (Fernandes et al. 2018), and AMPK activity (Tian et al. 2023). Additionally, it improves hippocampal size and cholinergic function at molecular, cellular, and structural levels (Fernandes et al. 2018). High‐intensity interval training (HIIT) is a type of exercise that includes intervals (from 45 s to 4 min) of high‐intensity activity (i.e., > 85% max heart rate) and low‐intensity activity (∼50% of maximal heart rate) (Costigan et al. 2015). Compared to other forms of exercise, HIIT induces chronic physiological adaptations within a shorter time frame (Leite et al. 2021; NÍ Chéilleachair et al. 2017). Research indicates that HIIT can improve both cognitive function and mental health (NÍ Chéilleachair et al. 2017).

Considering the improvement effects of exercise on learning and memory, it may be suggested that HIIT could alleviate ethanol‐induced cognitive impairments. Because no previous study has investigated the combined effects of HIIT and RIT on memory function in ethanol‐exposed animals, the present study aimed to evaluate this. Specifically, we examined the effects of 8 weeks of HIIT and RIT, both alone and in combination, on learning and memory impairments. These impairments were assessed using the Morris water maze (MWM) and passive avoidance tasks following chronic ethanol administration in male rats.

Furthermore, to better understand the role of oxidative stress in cognitive impairments, as well as the potential positive effects of HIIT and RIT in ethanol consumers, we focused on the hippocampus.

## 2 Methods and Materials

### 2.1 Animals

Fifty‐six male Wistar rats (aged 2 months, weighing 200–250 g) were obtained from the animal house of Kerman University of Medical Sciences (Kerman, Iran). The rats were housed under a 12‐h light–dark cycle at temperature of 23°C ± 2°C with free access to food and water.

### 2.2 Drugs

Ethanol (96%) was purchased from Hamon Teb. IRAN Company and diluted with 0.9% normal saline to obtain a 20% ethanol solution. Ethanol was administered orally at a dose of 3 mL/kg/day by gavage (Lamarão‐Vieira et al. 2019). RIT was purchased from Actover Co., IRAN, and solved in 0.9 % normal saline. It was administered intraperitoneally at a dose of 10 mg/kg (Khalid et al. 2020). All oral and injectable solutions were freshly prepared on the day of administration throughout the experiments.

### 2.3 HIIT Protocol

The details of the training protocol were based on the method described by Khoramipour et al. (2023). Prior to the training experiments, all rats were familiarized with a motorized treadmill. They ran on the treadmill at a speed of 8 m/min with no incline for 10–15 min/day, 5 days a week, for 2 weeks (Ebrahimnezhad et al. 2023). Following this habituation phase, the HIIT, ET + HIIT, RIT + HIIT, and ET + RIT + HIIT groups underwent an incremental running test to determine their maximum speed (*V*_max_). Subsequently, the HIIT protocol was implemented 5 days a week for 8 weeks (Rajizadeh et al. 2024). During the exercise sessions, the treadmill shock system was turned off to avoid stress to the rats.

### 2.4 Experimental Protocol

**FIGURE 1:**
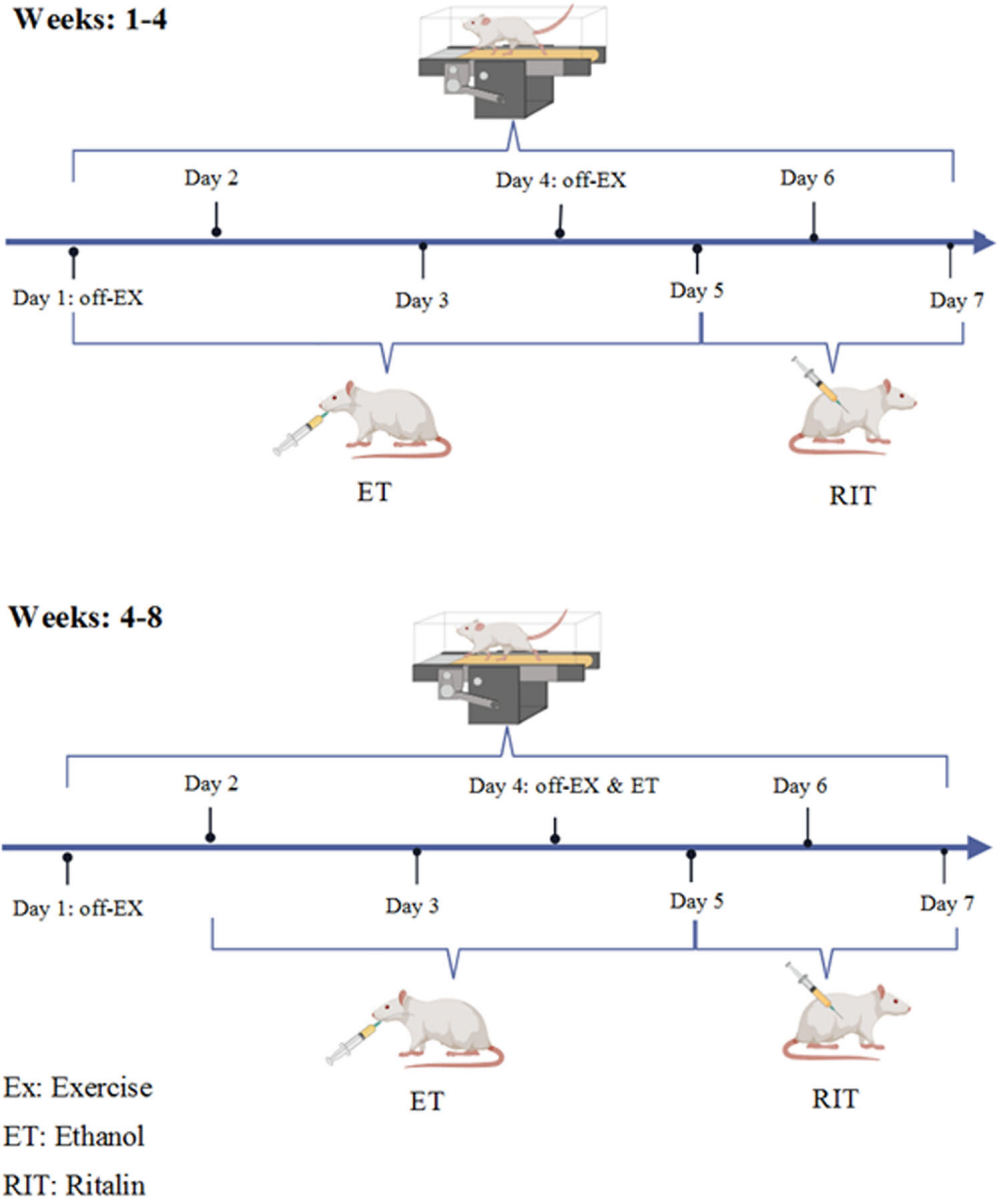
Timeline diagram.

To investigate the effects of HIIT and RIT on ethanol‐induced cognitive deficits, the rats were randomly divided into eight groups (*n* = 7 per group) as follows (Figure 1):

Group SAL: Rats received saline (1 mL/day, intraperitoneally, three consecutive times/week for 8 weeks).
Group ET: Rats received ethanol 20% for 8 weeks (3 mL/kg/day, orally, 5 consecutive days/week during weeks 1–4 and 3 consecutive days/week in weeks 4–8).
Group RIT: Rats received RIT (10 mg/kg, intraperitoneally, three consecutive times/week for 8 weeks).
Group HIIT: Rats underwent HIIT (5 consecutive days/week for 8 weeks) and received saline (1 mL/day, intraperitoneally, five consecutive times/week for 8 weeks).
Group ET + RIT: Rats received ethanol 20% (3 mL/kg/day, orally, 5 consecutive days/week during weeks 1–4 and 3 consecutive days/week in weeks 4–8) and RIT (10 mg/kg, intraperitoneally, 3 consecutive times/week for 8 weeks).
Group ET + HIIT: Rats received ethanol 20% (3 mL/kg/day, orally, 5 consecutive days/week during weeks 1–4 and 3 consecutive days/week in weeks 4–8) and underwent HIIT (5 consecutive days/week for 8 weeks).
Group RIT + HIIT: Rats received RIT (10 mg/kg, intraperitoneally, three consecutive times/week for 8 weeks) and underwent HIIT (5 consecutive days/week for 8 weeks).
Group ET + RIT + HIIT: Rats received ethanol 20% (3 mL/kg/day, orally, 5 consecutive days/week during weeks 1–4 and 3 consecutive days/week in weeks 4–8), RIT (10 mg/kg, intraperitoneally, three consecutive times/week for 8 weeks), and underwent HIIT (5 consecutive days/week for 8 weeks).

The timeline of treatments is illustrated in Figure 1. The MWM and passive avoidance tasks were conducted on separate days following the completion of treatments. All experiments were performed between 8:00 a.m. and 2:00 p.m. for all groups.

### 2.5 Behavioral Assessments

#### 2.5.1 Morris Water Maze (MWM)

The MWM test was used to assess spatial learning and memory in animals. The maze consisted of a dark circular pool (160 cm in diameter and 80 cm in height) filled with water to a depth of 40 cm (25°C ± 2°C). Geographically, the pool was divided into four equal quadrants (N, S, E, and W), and a black square platform (10 × 10 cm^2^) was hidden 1.5 cm beneath the water's surface in the center of the northeast quadrant. The MWM tests were performed in a dimly light room with several visual cues fixed on the walls surrounding the maze. Animal performance was recorded using a camera positioned above the pool. The Noldus Ethovision VR system (version 7.1, the Netherlands) was used to measure parameters.

The animals underwent three testing blocks to assess their spatial learning. Each block consisted of four sequential trials, each lasting 60‐s (inter‐trial interval = 30 s). During each block, animals were allowed to search for the hidden platform within the 60‐s trial. In each trial, the animals were placed in the pool at one of the geographical locations, facing the wall. The animal was allowed to swim and find the hidden platform within the 60‐s interval four times, whereas the platform's location was constant during the test. If the animal found the platform, it was allowed to stay on it for 20 s. The animal was then returned to its cage for 20 s before the next trial began. If the animal could not find the platform within 60 s, the experimenter guided it to the platform. Time spent, distance traveled to find the hidden platform, and velocity were recorded and analyzed in each trial (Taheri et al. 2022).

Two hours after the learning phase, a probe trial was conducted to evaluate long‐term spatial memory. In this phase, the platform was removed, and each animal was allowed to swim for 60 s. The time spent and distance traveled in the target quadrant (the quadrant where the platform was located during the training phase) were measured to assess spatial memory. Following this phase, a visible platform test was performed to evaluate the animal's sensory and motor coordination or motivation. For this test, the platform was placed 2 cm above the water level, covered with aluminum foil to make it visible, and the animal's ability to locate the visible platform was assessed (Taheri et al. 2019).

#### 2.5.2 Passive Avoidance Test

The passive avoidance test was used to evaluate associative learning and memory in rodents. A shuttle‐box apparatus was employed, with dimensions of 100 cm (length) × 25 cm (width) × 25 cm (height), consisting of two compartments (light and dark) separated by a sliding door. This task included three phases:

**Habituation**: Each rat was placed in the light chamber and allowed to move freely into the dark chamber for 5 min. Rats that did not enter the dark chamber were excluded from the experiment.

**Learning phase**: Two hours after habituation, each rat was placed in the light chamber; the door was opened after 10 s, and the door to the dark chamber was opened. Upon entering the dark chamber, the rat received an electric shock (50 Hz, 0.5 mA, 2 s) delivered via wires embedded in the floor of the dark chamber. This procedure was repeated at 2‐min intervals until the rat learned to avoid the dark chamber (remained in the light chamber for at least 120 s). The number of shocks received was recorded as an index of learning.

**Memory phase**: Memory was assessed 24 h after the learning phase. The rat was placed in the light chamber with the door closed. After 10 s, the door was opened, and the time taken by the rat to enter the dark chamber was recorded as the step‐through latency (STL). After the door opened, the total time spent in the dark chamber during a 5‐min period was also recorded as a memory index (Taheri et al. 2023).

All behavioral experiments were conducted 1 day after the last administration of saline, RIT, and HIIT.

### 2.6 Hippocampus Tissue Dissection

After behavioral tasks, the animals were euthanized following deep anesthesia (exposure to CO_2_ atmosphere). After removing the animals’ brains, the hippocampus was carefully separated on ice. The tissue was homogenized, and the homogenized tissue was centrifuged at 14,000 r/min at 4°C for 20 min.

### 2.7 Hippocampus Oxidative Stress Status Assessment

Malondialdehyde (MDA) compound measured as a lipid peroxidation index. The MDA level was measured by the related kit via the thiobarbituric acid reactive substances (TBARS) method (Abolhassani et al. 2019; Bejeshk et al. 2023). The total antioxidant capacity (TAC) level was measured via the ferric reduction antioxidant power (FRAP) method using spectrophotometry and a specific kit (Benzie and Strain 1996; Rajizadeh et al. 2023b). The GPX activity was measured by specific related kit (Rajizadeh et al. 2023a). Griess method was used to measure the levels of nitric oxide (NO) in serum (Yucel et al. 2012).

### 2.8 Statistical Analysis

The data are presented as mean ± SEM. Before selecting the analysis of variance (ANOVA) test, the homogeneity of variances was examined using Brown–Forsythe test, which confirmed that variances were homogeneous among groups (*p* > 0.05). The Shapiro–Wilk normality test was used to assess the normality of the data distribution. Parametric statistics were applied for data showing normal distribution, whereas the Kruskal–Wallis test was used for nonparametric data.

A repeated‐measure two‐way ANOVA was performed to determine differences in the learning (groups and blocks as the factors). One‐way ANOVA was employed for the MWM probe trial, step‐through latency phase of passive avoidance, and oxidative stress results. The Kruskal–Wallis was used to analyze the number of shocks and time spent in the dark chamber in the passive avoidance test. When statistical significance was observed among the groups, Tukey's multiple comparison test was conducted post hoc. A *p* value < 0.05 was considered statistically significant. Statistical analyses were performed using GraphPad Prism 8.0 software (GraphPad Software Inc., San Diego, CA).

## 3 Results

### 3.1 Effects of RIT and HIIT on Spatial Learning and Memory Following Ethanol Administration in the MWM

**FIGURE 2:**
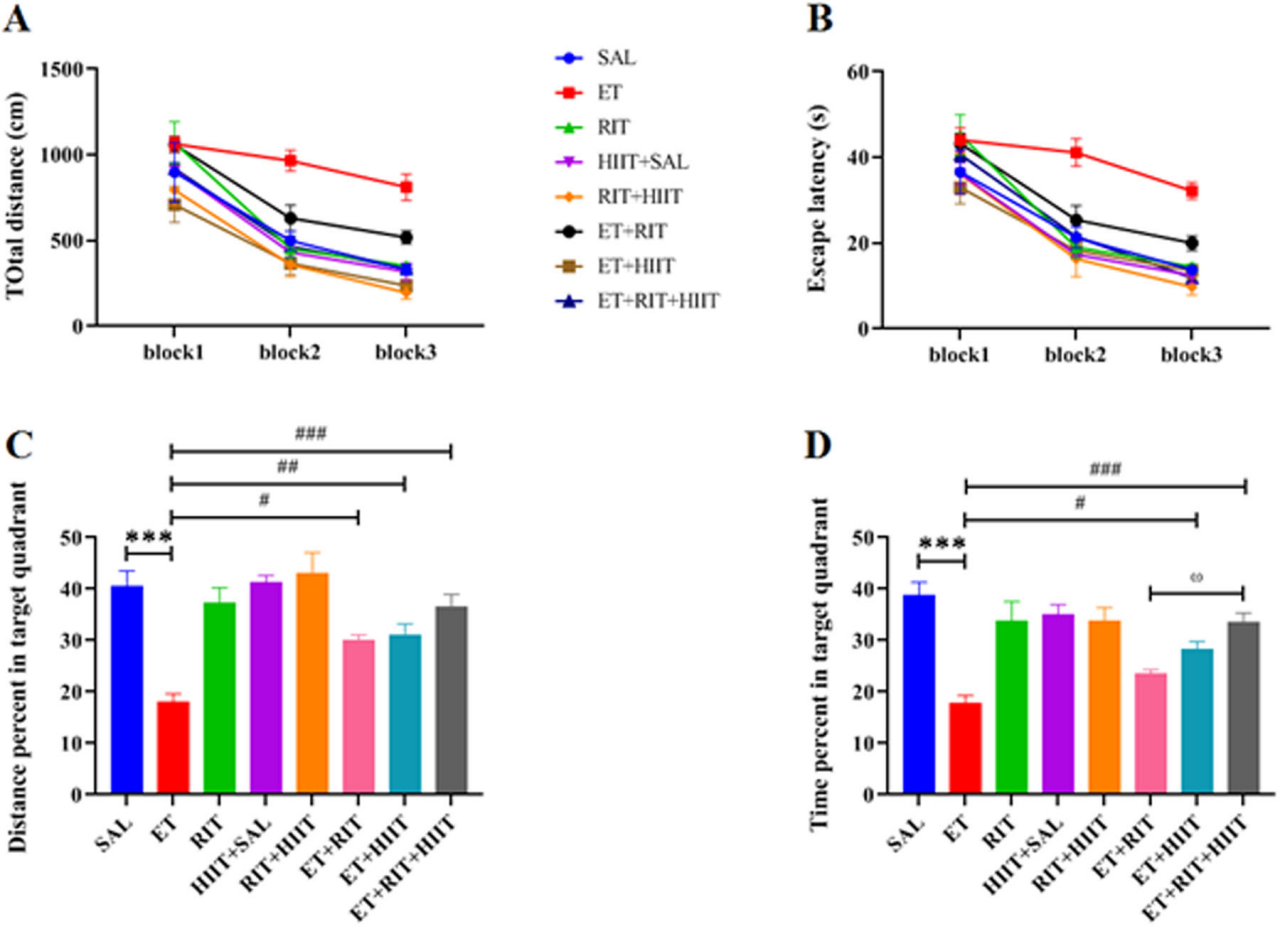
Impacts of RIT and HIIT on the performance of study groups in the MWM. (A) Total distance moved was increased in the ET group compared to the SAL group. However, total distance moved was reduced in the ET + RIT, ET + HIIT, and ET + RIT + HIIT groups compared to the ET group. Furthermore, the combination of Ritalin and HIIT decreased total distance in the ET + RIT + HIIT group compared to the ET + RIT group (B). Escape latency onto the hidden platform was increased in the ET group compared to the SAL group. However, escape latency was reduced in the ET + RIT, ET + HIIT, and ET + RIT + HIIT groups compared to the ET group. Interestingly, the combination of Ritalin and HIIT decreased total distance in the ET + RIT + HIIT group compared to the ET + RIT group (two‐way ANOVA, repeated measures). (C) Distance percent in the target quadrant was reduced in the ET group compared to the SAL group. However, distance percent increased in the ET + RIT, ET + HIIT, and ET + RIT + HIIT groups compared to the ET group (one‐way ANOVA). (D) Time percent in the target quadrant in the ET group was reduced compared to the SAL group. However, it was significantly increased in the ET + HIIT and ET + RIT + HIIT groups compared to the ET group. Additionally, the combination of Ritalin and HIIT increased time percent in the ET + RIT + HIIT group compared to the ET + RIT group (one‐way ANOVA). Data are reported as mean ± SEM. ٭٭٭*p *< 0.001 versus SAL, #*p *< 0.05, ##*p *< 0.01, ###*p *< 0.001 versus ET, ^ω^ *p *< 0.05 versus ET + RIT. ANOVA, analysis of variance; HIIT, high‐intensity interval training; MWM, Morris water maze; RIT, Ritalin.

The MWM was used to assess spatial learning and memory in the current study. Learning was defined as a decrease in swimming path length and escape latency to find the hidden platform during training phase. Data analysis revealed significant differences among groups in the path length (*F* [7, 48] = 11.57, repeated‐measure two‐way ANOVA). The total distance was increased in the ET group compared to the SAL group (Block 2: *p* < 0.01 & Block 3: *p* < 0.01) (Figure 2A). Total distance was reduced in the ET + HIIT and ET + RIT + HIIT groups compared to the ET group in Block 2 (ET + HIIT: *p* < 0.001 & ET + RIT + HIIT: *p* < 0.01). Furthermore, a significant reduction in total distance was observed between ET + HIIT and ET + RIT + HIIT groups compared to the ET group in Block 3 (*p* < 0.01). Moreover, the combination of RIT and HIIT decreased total distance in the ET + RIT + HIIT group compared to the ET + RIT group (*p* < 0.05) (Figure 2A).

Significant differences were observed in escape latency among groups (*F* [7, 48] = 10.46, repeated‐measure two‐way ANOVA). Ethanol increased escape latency in ET group in Blocks 2 and 3 of the learning test (Blocks 2: *p* < 0.01 & Blocks 3: *p* < 0.001). Additionally, lower escape latency was observed in the ET + HIIT and ET + RIT + HIIT groups compared to the ET group in Block 2 (*p* < 0.01), and in the ET + RIT, ET + HIIT, and ET + RIT + HIIT groups compared to the ET group in Block 3 (ET + RIT: *p* < 0.01 & ET + HIIT and ET + RIT + HIIT: *p* < 0.001). Interestingly, the combination of RIT and HIIT decreased total distance in the ET + RIT + HIIT group compared to the ET + RIT group (*p* < 0.05) (Figure 2B).

Two hours after learning phase, the probe test was used to assess spatial memory, and the mean percentage of distance traveled and time spent in the target quadrant (distance and time percents) were analyzed. A significant difference in the distance traveled was observed among all groups (*F* [7, 48] = 11.74, one‐way ANOVA). Our findings revealed a reduction in the distance traveled in the target quadrant in the ET group compared to the SAL group (*p* < 0.001). However, the distance traveled increased in the ET + RIT, ET + HIIT, and ET + RIT + HIIT groups compared to the ET group (ET + RIT: *p* < 0.05, ET + HIIT: *p* < 0.01, and ET + RIT + HIIT: *p* < 0.001) (Figure 2C).

Additionally, a significant difference was observed in the time percent among all groups (*F* [7, 48] = 10.65). The ET group spent less time in the target quadrant compared to the SAL group (*p* < 0.001). No significant difference was observed in the time percent in the target quadrant between the ET and ET + RIT groups. HIIT increased the time percent in the target quadrant in the ET + HIIT group compared to the ET group (*p* < 0.05). Furthermore, the combination of HIIT and RIT increased the time percent in the target quadrant in the ET + RIT + HIIT group compared to the ET group (*p* < 0.001). Moreover, the combination of RIT and HIIT increased the time percent in the ET + RIT + HIIT group compared to the ET + RIT group (*p* < 0.05) (Figure 2D).

Data analysis indicated no significant differences in swimming speed or latency to find the visible platform among the groups. Our manipulations did not affect the motor or sensory abilities of the experimental animals (Table 1).

**TABLE 1:** A is velocity, and B is visible test data.

A		B
Velocity (cm/s)		Time to find the platform(s)
Group	Mean ± SEM	Mean ± SEM
SAL	22.01 ± 0.85	18.93 ± 1.31
ET	19.81 ± 0.81	21.03 ± 2.14
RIT	20.65 ± 0.69	19.85 ± 1.93
HIIT	23.45 ± 0.61	18.08 ± 0.96
RIT + HIIT	21.97 ± 0.66	19.81 ± 2.67
ET + RIT	21.61 ± 0.71	21.93 ± 1.98
ET + HIIT	21.32 ± 0.53	19.98 ± 1.66
ET + RIT + HIIT	22.47 ± 1.06	22.38 ± 1.92

*Note*: No significant difference was observed in swimming speed and latency to find the visible platform among experimental groups.

Abbreviations: HIIT, high‐intensity interval training; RIT, Ritalin.

### 3.2 Effects of RIT and HIIT on Passive Avoidance Learning and Memory Following Ethanol Administration in the Passive Avoidance Test

**FIGURE 3:**
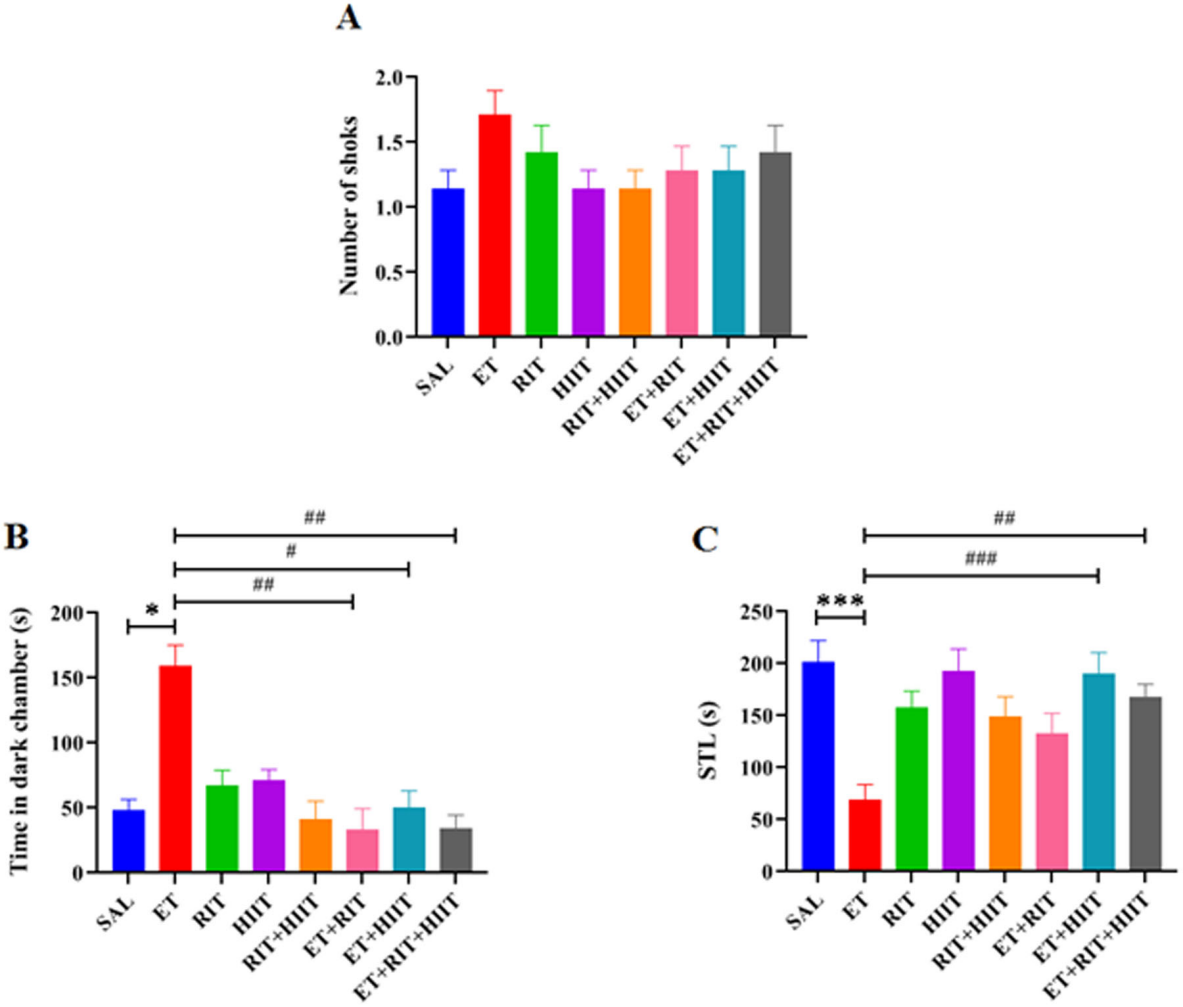
The impacts of RIT and HIIT on the learning trial (shock number) (A) and retention test, including time spent in the dark chamber (B) and step‐through latency (C). (A) There was no significant difference in the number of shocks between the ET and SAL groups. (B) Time spent in the dark compartment was significantly increased in the ET group compared to the SAL. However, Ritalin and HIIT reduced time spent in the dark chamber in the ET + RIT, ET + HIIT, and ET + RIT + HIIT groups compared to the ET group. (C) Step‐through latency was significantly reduced in the ET group compared to the SAL. Furthermore, Ritalin and HIIT increased time spent in the dark chamber in the ET + HIIT and ET + RIT + HIIT groups compared to the ET group. One‐way ANOVA and Kruskal–Wallis were used for statistical analysis. Data are reported as mean ± SEM. *n* = 7. ٭*p* < 0.05, ٭٭٭*p *< 0.001 versus SAL, #*p* < 0.05, ##*p* < 0, ###*p *< 0.001 versus ET. ANOVA, analysis of variance; HIIT, high‐intensity interval training; RIT, Ritalin.

The Kruskal–Wallis test showed no significant difference in the number of shocks between groups (Figure 3A). Figure 3B,C displays the effects of RIT and HIIT on passive avoidance memory. Time spent in the dark compartment increased in the ET group compared to the SAL group (*p* < 0.05, Kruskal–Wallis). However, RIT and HIIT reduced time spent in the dark compartment in the ET + RIT, ET + HIIT, and ET + RIT + EHIIT groups compared to the ET group (ET + HIIT: *p* < 0.05, ET + RIT & ET + RIT + HIIT: *p* < 0.01). No significant differences were observed between ET + RIT and ET + HIIT groups compared to the ET + RIT + HIIT group (Figure 3B).

The one‐way ANOVA test showed STL was significantly reduced in the ET group compared to the SAL group (*p* < 0.001). Additionally, STL significantly increased in the ET + HIIT and ET + RIT + HIIT groups compared to the ET group (ET + HIIT: *p* < 0.001 and ET + RIT + HIIT: *p* < 0.01) (Figure 3C).

### 3.3 Effects of RIT and HIIT on the Oxidative Status Following Ethanol Administration in the Hippocampus

**FIGURE 4:**
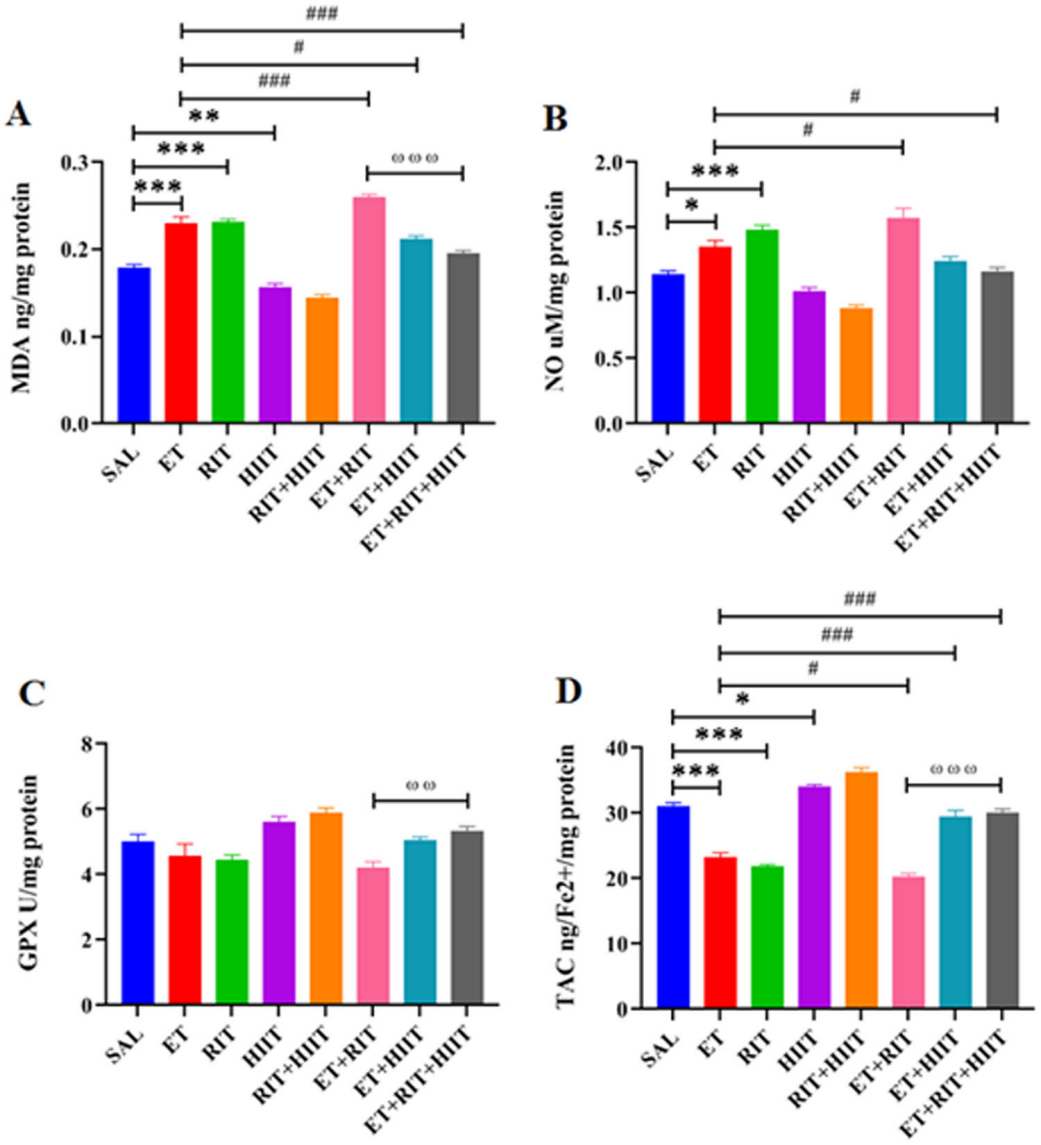
The impacts of RIT and HIIT on the oxidative status (MDA) (A), (NO) (B), (GPx) (C), and (TAC) (D). (A) Ethanol and Ritalin increased MDA levels, whereas HIIT decreased MDA levels. (B) Furthermore, ethanol and Ritalin increased NO levels, whereas HIIT decreased NO levels in the ET + RIT + HIIT group compared to the ET group. (C) Ritalin decreased GPx activity in the ET + RIT group compared to the ET + RIT + HIIT group. (D) Ethanol and Ritalin decreased TAC levels, whereas HIIT increased TAC levels. One‐way ANOVA was used for statistical analysis. Data are reported as mean ± SEM. *n* = 4. ٭*p* < 0.05, ٭٭٭*p* < 0.001 versus SAL, #*p* < 0.05, ##*p* < 0.01, ###*p* < 0.001 versus ET, ^ωω^ *p* < 0.01, ^ωωω^ *p* < 0.001 versus ET + RIT + HIIT. ANOVA, analysis of variance; GPx, glutathione peroxidase; HIIT, high‐intensity interval training; MDA, malondialdehyde; MWM, Morris water maze; NO, nitric oxide; RIT, Ritalin; TAC, total antioxidant capacity

The ET and RIT groups displayed a significant increase in MDA levels (*p* < 0.001) compared to SAL levels (Figure 4A). In contrast, a significant reduction in MDA levels (*p* < 0.001) was observed in the HIIT + SAL group compared to the SAL group. Additionally, the ET + RIT group showed a significant increase in MDA levels compared to the ET group (*p* < 0.001), whereas HIIT decreased MDA levels in the ET + HIIT and ET + RIT + HIIT groups (ET + HIIT: *p* < 0.05 and ET + RIT + HIIT: *p* < 0.001) (Figure 4A). Furthermore, a significant increase in MDA levels was observed in the ET + RIT group (*p* < 0.001) compared to the ET + RIT + HIIT group (Figure 4A).

Ethanol and RIT increased NO levels in the ET and RIT groups (ET: *p* < 0.05 and RIT: *p* < 0.001, one‐way ANOVA) versus SAL levels (Figure 4B). Additionally, RIT increased NO levels (*p* < 0.05) in the ET + RIT group compared to the ET group, whereas NO levels decreased in the ET + RIT + HIIT group compared to the SAL group (*p* < 0.05) (Figure 4B). No significant differences were observed in NO levels in the ET + RIT and ET + HIIT groups compared to the ET + RIT + HIIT group (Figure 4B).

No significant differences were observed in glutathione peroxidase (GPx) activity among the groups (Figure 4C). However, a significant decrease in GPx activity was found in the ET + RIT group compared to the ET + RIT + HIIT group (*p* < 0.01) (Figure 4C).

A reduction in TAC levels was observed in the ET and RIT groups compared to the SAL group (*p* < 0.001). Notably, HIIT increased TAC levels in the HIIT + SAL (*p* < 0.05). RIT reduced TAC levels in the ET + RIT group compared to the ET group (*p* < 0.05). Furthermore, HIIT enhanced TAC levels in the ET + HIIT and ET + RIT + HIIT groups compared to the ET group (*p* < 0.001, one‐way ANOVA). TAC levels were reduced in the ET + RIT group compared to the ET + RIT + HIIT group (*p* < 0.001) (Figure 4D).

## 4 Discussion

The current study is the first to demonstrate the effect of HIIT and RIT, alone and in combination, on cognitive function and oxidative stress markers following ethanol administration in male rats. Cognitive impairments and increased oxidative stress were observed in rats following ethanol administration; interestingly, HIIT improved these deficits. However, RIT showed beneficial effects only on cognitive behavior, not on oxidative stress.

In our study, the MWM was used to evaluate the rats’ spatial learning and memory. Our findings revealed that ethanol administration caused spatial learning and memory impairments in animals. Ethanol increased the total distance traveled and escaped latency to find the hidden platform while decreasing the distance traveled and time spent in the target quadrant during the memory phase of the MWM. These findings support previous studies, which also reported that long‐term ethanol consumption causes cognitive impairments, including deficits in learning and memory (Akbari et al. 2023; Li et al. 2019; Hasanein et al. 2017; Zorumski et al. 2014).

Passive avoidance is a fear‐aggravated task used to assess learning and memory in rodents. In this test, animals learn to avoid an environment where an aversive stimulus (such as a foot shock) is delivered. Our results demonstrated that ethanol administration increased the time spent in the dark compartment and reduced step‐through latency in the passive avoidance test in rats. These findings align with previous studies showing that chronic ethanol consumption and late periods of ethanol withdrawal impair performance in the passive avoidance task (Çelik et al. 2005).

Ethanol‐induced learning and memory impairments are mediated by several mechanisms, including oxidative damage to the CNS and alteration in glutamate (Glu) and γ‐aminobutyric acid (GABA) levels in the hippocampus (Li et al. 2019; Hasanein et al. 2017; Zorumski et al. 2014; Soleimani et al. 2016). The beneficial effects of natural antioxidants in ameliorating ethanol‐induced learning and memory deficits have been reported in several studies (Mahdinia et al. 2021; Zhang et al. 2015).

Current findings indicate that RIT administration ameliorated spatial learning and memory deficits in ethanol‐exposed rats. RIT decreased total distance and escape latency to find the hidden platform and enhanced the distance traveled in the target quadrant during the memory phase. These results are consistent with previous studies that demonstrated the beneficial effects of RIT on spatial memory (Motamedi et al. 2019; Khalid et al. 2020). Furthermore, RIT improved ethanol‐induced memory deficits in the passive avoidance task by decreasing the time spent in the dark compartment after its administration. Studies have suggested that RIT enhances synaptic transmission, ultimately improving memory (Khalid et al. 2020).

Several investigations have revealed the positive effects of exercise on learning and memory in humans and animals (Khodadadi et al. 2018; Yin et al. 2013; Mu et al. 2022; Zang et al. 2023; Cassilhas et al. 2016). Roig et al. (2013) reported the beneficial impacts of acute, but not long‐term, exercise on memory improvement in a time‐dependent manner by priming molecular processes involved in encoding and consolidating newly acquired data. Exercise carried out 4 h was associated with improving memory retention, suggesting long‐term memory improvement following appropriately timed physical exercise (van Dongen et al. 2016). Our findings support previous studies and reveal that HIIT, like other forms of exercise, ameliorated learning and memory deficits in ethanol‐exposed animals. HIIT reduced total distance and escape latency to find the hidden platform while increasing the distance traveled and time spent in the target quadrant.

Additionally, our findings indicate that HIIT reversed impairments in passive avoidance memory. Consistent with our results, studies have shown that treadmill exercise enhances passive avoidance memory by downregulating serotonin in the limbic system (Chen et al. 2008). Moreover, the combination of HIIT and RIT had more beneficial effects on spatial learning and memory deficits than RIT alone in the MWM test. Exercise positively affects cognitive behavior through several mechanisms, including the enhancement of BDNF release.

BDNF release is a crucial mechanism underlying the improvement of cognitive function after exercise (Erickson et al. 2019; Walsh et al. 2020). Exercise influences the function of BDNF and its specific receptor, tropomyosin receptor kinase B (TrkB), which are critically involved in synaptic plasticity and learning and memory processes (Xu et al. 2021; Ahmadalipour et al. 2018). Previous studies have shown that physical exercise enhances neurogenesis and improves memory (Kim et al. 2010). Moreover, exercise can ameliorate memory by increasing norepinephrine and dopamine release into the synaptic cleft (Veening and Barendregt 2015) and the number of new hippocampal cells (Biedermann et al. 2016). Furthermore, clinical studies have revealed that after a 6‐week exergaming training, the volumes of CA1, CA4, and DG were significantly increased in the left hippocampus in patients with Parkinson's disease (Schaeffer et al. 2022).

We also investigated oxidative stress factors in the hippocampus of rats as a possible mechanism. We showed that both ethanol‐ and RIT‐treated animals exhibited a significant enhancement in MDA and NO levels and a significant reduction in TAC levels in the hippocampus versus the saline‐treated rats. Furthermore, HIIT exercise was associated with a significant decrease in MDA level and a significant increase in TAC in the rat's hippocampus region. Our findings revealed that HIIT increased MDA and TAC levels as well as decreased NO levels in present RIT and ethanol in the ET + RIT + HIIT group. In fact, HIIT could reverse effects of ethanol and RIT on oxidative stress. These findings supported previous studies indicating that physical exercise alleviates ethanol‐induced oxidative stress in rats (Patil et al. 2015; Soleimani et al. 2016; Hernández et al. 2016). Consistent with our findings, other investigators reported that physical exercise alleviates the oxidative stress caused by ethanol consumption in rats (Lamarão‐Vieira et al. 2019; Brocardo et al. 2012; Pamplona‐Santos et al. 2019).

Several investigations have revealed similar effects of RIT that create oxidative and inflammatory alterations, leading to neuronal damage, particularly degeneration of dopaminergic neurons (Motaghinejad et al. 2017; Thomas et al. 2004). Previous studies have revealed that RIT induces the expression of inflammatory cytokines, such as tumor necrosis factor alpha (TNF‐α) and interleukin‐1β (IL‐1β), resulting in cognitive impairments (Motaghinejad, Motevalian, Falak et al. 2016; Motaghinejad, Motevalian, and Shabab 2016).

Our results showed that ethanol probably induces cognitive impairment, whereas HIIT improves these deficits by moderating oxidative stress in the brain. Possibly, RIT improves memory function through other mechanisms in the brain. Studies have demonstrated that RIT improves cognitive function by preventing the reuptake of dopamine and norepinephrine in the synaptic cleft (Oakes et al. 2019). Clinical studies have reported the improving effects of RIT on BDNF plasma concentrations in children with attention deficit hyperactivity disorder (ADHD) (Amiri et al. 2013). RIT might also impact neuronal survival by modulating apoptosis. It is able to modulate apoptosis‐related proteins, including apoptosis regulator Bcl‐2 (BCL2), BAX (BAX), and caspase‐3 (CASP3) (Réus et al. 2014). RIT indirectly activates dopamine postsynaptic receptors (Ko et al. 2019) that uptake dopamine and transmit the signal in postsynaptic neurons (Gronier 2011).

It seems that HIIT is considered an effective treatment for cognitive impairment, especially following ethanol administration in rats. Because it is able to act on brain function by several mechanisms, including reducing apoptosis. In fact, treadmill exercise has been shown to suppress CASP3 expression in the hippocampus (Choi et al. 2013; Kim et al. 2014), increase B‐cell lymphoma 2 (Bcl‐2), and decrease Bcl‐2‐associated X (Bax) expression (Park et al. 2020). Furthermore, long‐term treadmill exercise improves spatial memory performance by decreasing TNF‐α, IL‐6, and IL‐1β (Kim et al. 2021). Exercise has also been shown to increase dendritic complexity and the number of dendritic spines in the dentate gyrus, leading to better cognitive performance (Lee et al. 2021). Meanwhile, Li et al. (2021) reported that adaptive treadmill training for 12 weeks, 5 days/week, 45 min a day, led to improved spatial learning and memory in mice, with significant increases in the number of synapses in the CA1 region of the hippocampus.

On the basis of the above findings and our results, it seems that HIIT exercise can probably serve as a replacement therapy for cognitive impairments in ethanol consumers instead of drug therapy. Although more molecular and histological investigations are needed in future studies.

This study has several limitations that should be taken into account in future research. We were unable to include female rats in our study due to financial constraints. Additionally, the role of other brain regions and systems remains unclear. Further research, particularly at the molecular and histological levels, is necessary to better understand the underlying mechanisms of the beneficial effects of HIIT in ethanol administration.

## 5 Conclusion

Chronic ethanol consumption caused learning and memory deficits and disrupted hippocampal oxidant/antioxidant balance in rats. This study demonstrated for the first time that HIIT improved memory impairments by restoring oxidant/antioxidant balance, whereas RIT ameliorated cognitive dysfunction through alternative mechanisms. These findings suggest that HIIT could be considered a potential replacement therapy for cognitive impairments associated with ethanol consumption. Further molecular and histological investigations are needed to explore the underlying mechanisms.

## Author Contributions

**Sara Shirazpour**: writing–review and editing, writing–original draft. **Farahnaz Taheri**: writing–review and editing, writing–original draft. **Gholamreza Sepehri**: investigation, supervision. **Mahla Zangiabadizadeh**: methodology. **Mostafa Zangiabadi**: investigation. **Najmeh Sadat Hosseini**: methodology. **Sara Sheikhi**: investigation. **Azadeh Shahrokhi Raeini**: methodology. **Sara Sheibani Tezerji**: formal analysis.

## Ethics Statement

All experimental procedures were conducted in accordance with the guidelines for the care of experimental animals and were approved by the Institutional Animal Research Ethics Committee, Kerman University of Medical Sciences (Ethics code: IR.KMU.AEC.1401.005).

## Conflicts of Interest

The authors declare no conflicts of interest.

### Peer Review

The peer review history for this article is available at https://publons.com/publon/10.1002/brb3.70539

## Data Availability

The data that support the results of the current study are available from the corresponding author upon reasonable request.
